# War Babies and the Mother

**Published:** 1915-08

**Authors:** 

**Affiliations:** San Antonio


### War Babies and the Mother.

It is a fact that in the present European war men and women have been encouraged to marry that more children may be born to replenish the loss of the fallen in battle. It is also a fact now known, that many of these children will be illegitimate in sight of the law; and, it now becomes a problem as to the proper treatment to be accorded them. The old stigma of immorality which is attached to the meaning of illegitimacy must be in some measure qualified. Public sentiment is fast changing in regard to the eugenic relation of woman and the child to society. Who now would contend that the unborn child should not have the right of an equal chance, morally and physically, with every other child? Who is responsible for the child's birth? Not the child. Then, if he is not responsible for his existence he should not be made to bear the handicap that society and the State imposes on him by the stigma of illegitimacy. No amount of penitence and suffering of the mother, imposed by moral discipline, can atone for this injustice to the child or in any manner mitigate his wrong. By inflicting disabilities on the child you not only deny him his rightful opportunity to develop his best heritage but by so doing you force him to become a charge on society. Therefore it follows that the State has the moral and social obligation to correct this social wrong. It is simply a matter of adjusting our inherited ideas of morality to fit present conditions in the light of our deeper understanding of humanity and justice. To those who would further quibble over the "moral" aspect of the situation, let them answer this question. Which is the most immoral, murder or adultery? We are commanded not to kill and not to commit adultery. Who, then, can sanction the recruiting of soldiers for wholesale murder and condemn the patriotic, though misguided, motive of the mother producing illegitimate children? Both are wrong! Yes, both *are* wrong. But so long as we must condone the necessity of war we must be equally charitable toward the other *wrong*.

What we need is more enlightenment on the real and supposed rights of the child and its mother. The only standard by which we can judge what is moral is that conduct which insures the greatest happiness and success for the individual and the race as a whole. Judging by this standard it would be extremely immoral to advocate family extension during the war, as it means the birth of children under economic conditions utterly inappropriate to the best needs of the child and adds burdens to the mother that is more inhuman than the suffering and death experienced by the father on the battlefield.

Another feature to the question is the ethical relation of woman to the race. While she does not sacrifice her life on the battlefield she *does* give her best to replenishing the people who must be so sacrificed. Then, why should she be held in account for exercising her pleasure or motive in the matter of maternity? Indeed, why should she not be sustained and protected in the discharge of her maternal function? She is more to be revered than condemned, and, if perchance her sense of patriotism has led her in excess of the right of her unborn child she should have the support and protection of the State.

